# Novel homozygous variants in *TTC12* cause male infertility with asthenoteratozoospermia owing to dynein arm complex and mitochondrial sheath defects in flagella

**DOI:** 10.3389/fcell.2023.1184331

**Published:** 2023-06-01

**Authors:** Lanlan Meng, Qiang Liu, Chen Tan, Xilin Xu, Wenbin He, Tongyao Hu, Chaofeng Tu, Yong Li, Juan Du, Qianjun Zhang, Guangxiu Lu, Li-Qing Fan, Ge Lin, Hongchuan Nie, Huan Zhang, Yue-Qiu Tan

**Affiliations:** ^1^ NHC Key Laboratory of Human Stem Cell and Reproductive Engineering, School of Basic Medical Sciences, Institute of Reproductive and Stem Cell Engineering, Central South University, Changsha, Hunan, China; ^2^ Clinical Research Center for Reproduction and Genetics in Hunan Province, Reproductive and Genetic Hospital of CITIC-Xiangya, Changsha, Hunan, China; ^3^ Hunan Cancer Hospital and the Affiliated Cancer of Xiangya School of Medicine, Central South University, Changsha, Hunan, China; ^4^ College of Life Science, Hunan Normal University, Changsha, Hunan, China

**Keywords:** male infertility, *TTC12*, asthenoteratozoospermia, dynein arm complex defects, mitochondrial sheath malformations

## Abstract

**Introduction:** Tracing the genetic causes for male infertility due to asthenoteratozoospermia has revealed at least 40 causative genes, which provides valuable reference for the genetic testing of asthenoteratozoospermia in clinical practice. To identify deleterious variants in the human tetratricopeptide repeat domain 12 (TTC12) gene in a large cohort of infertile Chinese males with asthenoteratozoospermia.

**Methods:** A total of 314 unrelated asthenoteratozoospermia-affected men were recruited for whole exome sequencing. The effects of the identified variants were evaluated by *in silico* analysis, and confirmed by *in vitro* experiments. Intracytoplasmic sperm injection (ICSI) was used to evaluate the efficiency of assisted reproduction technique therapy.

**Results and Discussion:** Novel homozygous *TTC12* variants (c.1467_1467delG (p.Asp490Thrfs*14), c.1139_1139delA (p.His380Profs*4), and c.1117G>A (p.Gly373Arg)) were identified in three (0.96%) of the 314 cases. Three mutants were indicated to be damaging using *in silico* prediction tools, and were further confirmed by *in vitro* functional analysis. Hematoxylin and eosin staining and ultrastructural observation of the spermatozoa revealed multiple morphological abnormalities of flagella, with the absence of outer and inner dynein arms. Notably, significant mitochondrial sheath malformations were also observed in the sperm flagella. Immunostaining assays indicated that TTC12 is present throughout the flagella, and was strongly concentrated in the mid-piece in control spermatozoa. However, spermatozoa from *TTC12*-mutated individuals exhibited almost no staining intensity of TTC12 and outer and inner dynein arms components. The three men accepted ICSI treatment using their ejaculated spermatozoa, and two female partners successfully delivered healthy babies. Our findings provide direct genetic evidence that homozygous variants in *TTC12* cause male infertility with asthenoteratozoospermia by causing dynein arm complex defects and mitochondrial sheath malformations in the flagellar. We also demonstrated that TTC12 deficiency-mediated infertility could be overcome by ICSI technology.

## Introduction

Asthenoteratozoospermia, characterized by reduced sperm motility and increased sperm morphological abnormalities, is a common cause of male infertility (Krausz and Riera-Escamilla, 2018; Shahrokhi et al., 2020) and is suspected of contributing to approximately 19% of male infertility cases (Agarwal et al., 2015; Krausz and Riera-Escamilla, 2018). The spermatozoon flagellum is a motile apparatus consisting of a mid-piece, principal-piece, and end-piece. The axoneme is the core component of the spermatozoon flagellum, which is an evolutionarily conserved structure consisting of a 9 + 2 arrangement of nine peripheral doublets of microtubules and a central pair of microtubules. The axonemal dynein arms, comprising outer and inner dynein arms (ODAs and IDAs, respectively), are attached to the A-microtubule of each peripheral doublet of microtubules (Inaba, 2007; 2011).

Genetic variants have been widely recognized as the cause of a large proportion of sperm axoneme defect-related asthenoteratozoospermia cases (Coutton et al., 2015; Tu et al., 2020). Dynein-related genes, in particular, are emerging as new candidate genes, even for isolated cases of male infertility (Levkova et al., 2022). In the past few years, an increasing number of dynein-related genes linked to asthenoteratozoospermia have been identified in humans. For example, defects in two IDA heavy-chain proteins, namely, DNAH1 (Ben Khelifa et al., 2014) and DNAH2 (Gao et al., 2021), and two ODA heavy-chain components, namely, DNAH8 (Liu et al., 2020) and DNAH17 (Whitfield et al., 2019; Zhang et al., 2020; Zheng et al., 2021), have been described in human subjects with isolated male infertility due to asthenoteratozoospermia. In our previous studies, we identified several genes associated with asthenoteratozoospermia with abnormal axoneme structures, including *CFAP65* (Wang et al., 2019), *DNHD1* (Tan et al., 2022), *CFAP47* (Liu et al., 2021), and *DNAH10* (Tu et al., 2021). Most notably, variants in *DNHD1* and *CFAP65* also cause abnormalities in the mitochondrial sheath (MS).

Human tetratricopeptide repeat domain 12 (TTC12) is a recently-discovered gene involved in the assembly of ciliary and flagellar axonemes, and its biallelic variants have been reported to cause primary ciliary dyskinesia (PCD) in individuals from four separate families (Thomas et al., 2020). The symptoms included neonatal respiratory distress, rhinosinusitis, and bronchiectasis due to dysfunction of the motile cilia (Thomas et al., 2020). Two male adults carrying a homozygous missense variant [c.1700T > G (p.Met567Arg)] and a homozygous splice site variant [c.1614 + 3A > T (p.?)], respectively, also showed infertility due to asthenozoospermia (Thomas et al., 2020). However, the evidence for the association between TTC12 defects and asthenoteratozoospermia in the Chinese population has not been reported.

Our study identified homozygous *TTC12* deleterious variants in three patients from a cohort of 314 unrelated Han Chinese infertile men with asthenoteratozoospermia. The spermatozoon phenotypes of these three individuals included dynein arm complex defects and MS malformations. Our findings confirmed that *TTC12* variants play a causative role in the development of asthenoteratozoospermia. We also demonstrated that TTC12 deficiency-mediated infertility can be overcome by intracytoplasmic sperm injection (ICSI) technology.

## Materials and methods

### Study subjects

This study was approved by the ethics committee of CITIC Xiangya Reproductive Genetics Hospital (LL-SC-2017-025 and LL-SC-2019-034). We recruited 314 (Tan et al., 2022) Chinese of Han origin, diagnosed with idiopathic asthenoteratozoospermia according to the guidelines of the World Health Organization (WHO) laboratory manual (Cooper et al., 2010). Physical examinations, karyotype mapping, and Y-chromosome microdeletion tests were normal in all individuals. Hormone levels were normal for those individuals whose endocrinal status was necessary to evaluate according to Chinese experts’ consensus and the AUA/ASRM guideline for the diagnosis and treatment of infertility in men (Schlegel et al., 2021). Whether or not the collected cases had PCD-related symptoms, such as recurrent airway inflammation, bronchiectasis, and otitis media, was not an exclusion criterion. In this study, 392 healthy and fertile Chinese Han men with sperm concentrations ≥15 million/mL and sperm progressive activities ≥32% were recruited as a control.

### Semen parameter analysis

Semen routine was conducted according to WHO guidelines (Cooper et al., 2010). Ejaculated semen was obtained from infertile patients after 1 week of sexual abstinence, and semen volume, sperm concentration, and motility were calculated. The morphology of at least 200 sperm cells was examined by hematoxylin and eosin (H&E) staining to assess the percentage of morphologically abnormal spermatozoa.

### Whole-exome sequencing and verification

Genomic DNA (gDNA) from peripheral blood samples was extracted using the QIAamp DNA Blood Midi Kit (QIAGEN, 51106) according to the manufacturer’s protocol. The gDNA samples of the 314 infertile men were subjected to whole-exome sequencing (WES) on Illumina HiSeq 2000 or HiSeq X-TEN platforms (Tan et al., 2022). The WES data analysis was sequentially performed using the Burrows–Wheeler Aligner (BWA), Genome Analysis Toolkit (GATK), and ANNOVAR. Furthermore, candidate variants were filtered as previously described (Tan et al., 2019). The clinical significance of the identified variants was assessed following the standards and guidelines of the American College of Medical Genetics and Genomics (ACMG) (Richards et al., 2015). The verification of the candidate variants was conducted using the Green Mix kit (Promega, M7123) and target-site primers (Supplementary Table S1).

### Plasmid transfection and Western blotting

Full-length TTC12 was obtained from human cDNA by RT-PCR and inserted into the GV712 expression vector, leading to the production of fusion proteins with FLAG at the N-terminus of TTC12. Wild-type TTC12 plasmid was induced into three mutants (c.1467_1467delG, c.1139_1139delA, and c.1117G>A) by using the Mut Express II Fast Mutagenesis Kit V2 (Vazyme, C214) according to the manufacturer’s protocols. Wild-type and mutated TTC12 clones were confirmed by direct Sanger sequencing.

Human embryonic kidney cells (HEK293T) were cultivated at 37°C with 5% CO_2_ in six-well culture plates supplemented with 10% fetal bovine serum (Gibco, 10091-148). Wild-type TTC12 and three mutated plasmids were transiently transfected into HEK293T cells when the plate reached 60%–70% abundance using Lipofectamine 3000 (Invitrogen, L3000015) according to the manufacturer’s instructions. Cells were then harvested 48 h after transfection and homogenized with RIPA lysis buffer (Beyotime Biotechnology, P0013B) that was supplemented with Protease Inhibitor Cocktail (Thermo Scientific, 87786). Subsequently, the lysates were centrifuged (13,000 g, 15 min) at 4°C, and the supernatants were collected. Proteins were blotted onto polyvinylidene difluoride membranes and incubated overnight at 4°C with anti-FLAG (Abways, AB0008, 1:3,000) and anti-GAPDH antibodies (Abcam, ab8245, 1:3,000), respectively. The membranes were then incubated with secondary antibodies (goat anti-mouse IgG or goat anti-rabbit IgG, Abways, AB0101, 1:5,000). The blot results were detected using the ECL Western Blotting kit (Pierce Biotechnology).

### Scanning and transmission electron microscopy

Ejaculated semen samples from infertile patients and controls were washed three times with saline at 2000 rpm/min at room temperature and then fixed with 2.5% glutaraldehyde solution (pH 6.9) for more than 2 h at 4°C. For SEM, samples were sequentially fixed by dehydration using ascending gradient cold ethanol (50%, 70%, 95%, and 100%), dried at the critical point, coated with gold particles using an ion sputter coater and then observed using an S-3400N scanning electron microscope (Hitachi, Japan). For TEM, semen samples were fixed in glutaraldehyde and osmium tetroxide buffer, followed by dehydration through an ethanol gradient, and embedded in Epon 812. Sections were cut (70–90 nm) using a microtome, and then, ultrathin sections were stained with uranyl acetate and lead citrate. Images were taken using an HT7700 Hitachi electron microscope (Hitachi, Japan) and a MegaView III digital camera (Munster).

### Immunofluorescence assay

The immunofluorescence assay of the spermatozoa was performed as previously described (Tu et al., 2021). The antibodies used in this study are described in Supplementary Table S2.

### ICSI therapy

ICSI was conducted after signing of written informed consent. In brief, oocytes were retrieved from the female partner and were rinsed with 80 IU/mL of hyaluronidase 3–5 h later. Followed by the transfer of denuded oocytes into the G-MOPS medium (Vitrolife, 10130), spermatozoa with normal morphology and motility were transferred into polyvinylpyrrolidone (PVP) buffer and then injected into the selected oocyte by using a microinjection needle. The subsequent culture of the injected oocytes was conducted in the G-IVF medium using a humidified incubator with the following conditions: 37°C with the air condition of 6% CO_2_, 5% O_2_, and 89% N_2_.

## Results

### Identification of homozygous *TTC12* variants

**FIGURE 1:**
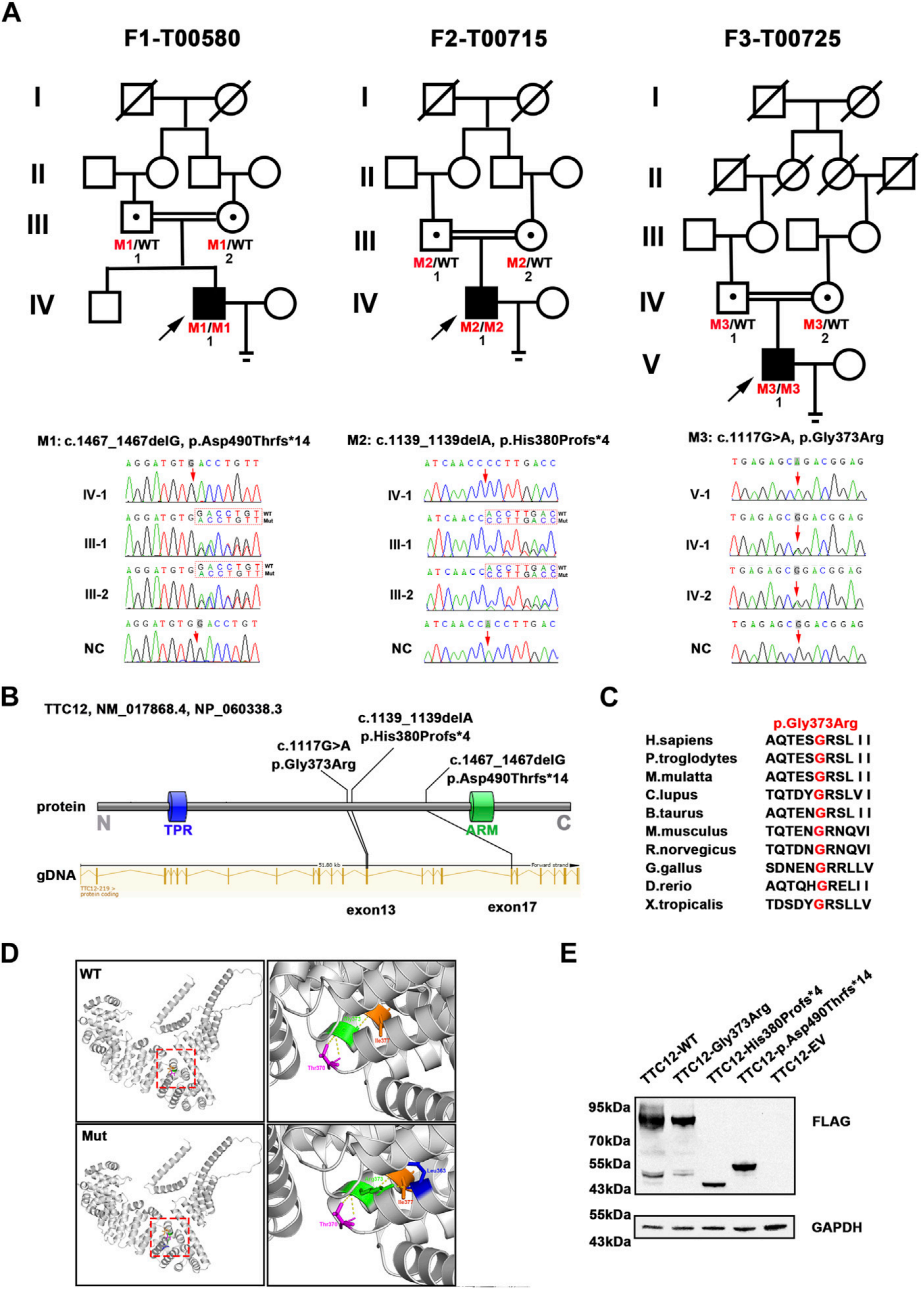
Identification of damaging *TTC12* variants in unrelated infertile men. **(A)** Pedigree analysis of three independent consanguineous infertile families with *TTC12* variants. Open signs were unaffected members. Open signs with a dot in the middle represented heterozygous carriers. Filled signs with black arrows indicated three probands. WT, wild type; NC, normal control. **(B)** Distribution of three TTC12 mutants at the protein and gDNA level. The blue squares in the TTC12 domain map represent the TRP repeat, and the green squares indicate ARM domains. All domains and regions are described using the NCBI gene server. **(C)** The residue Gly in the 373 locus is highly conserved among ten species according to the NCBI sequence alignment. **(D)** Structural model of WT and missense-mutated TTC12 proteins. The magnified views in the right panel show p.Gly373Arg mutant, which created a new hydrogen-bonding connection between Arg373 and Leu363 compared with the WT protein. The yellow dashed lines represent hydrogen bonds. **(E)** *In vitro* expression of TTC12 mutants in HEK293T cells. GAPDH was used as the internal protein.

According to the filtering criteria, WES identified three novel homozygous variants of *TTC12* (NM_017868.4: c.1467_1467delG, c.1139_1139delA, and c.1117G > A) from three unrelated consanguineous families, which accounted for 0.96% (3/314) of the asthenoteratozoospermia cohort in this study (Figure 1A; Table 1). Direct sequencing validated the homozygosity of c.1467_1467delG, c.1139_1139delA and c.1117G > A in our probands (F1-IV-1, F2-IV-1, and F3-V-1, respectively). The unaffected parents of these probands (F1-III-1 and F1-III-2, F2-III-1 and F2-III-2, and F3-IV-1 and F3-IV-2, respectively) were heterozygous carriers at the corresponding sites (Figure 1A), suggesting an autosomal recessive pattern for these *TTC12* variants.

**TABLE 1:** Clinical phenotype of three asthenoteratozoospermia-affected men with novel *TTC12* variants.

Patient ID	Gene	cDNA alteration	Protein alteration	Variant zygosity	Function	Gene allele frequency			*In silico* prediction			
						1000 Genomes	GnomAD	GnomAD-EAS	PolyPhen-2	MutationTaster	PROVEAN	CADD
F1-T00580-IV-1	*TTC12*	c.1467_1467delG	p.Asp490Thrfs*14	HOM	Frameshift	NA	NA	NA	—	D	—	—
F2-T00715-IV-1	*TTC12*	c.1139_1139delA	p.His380Profs*4	HOM	Frameshift	NA	NA	NA	—	D	—	—
F3-T00725-V-1	*TTC12*	c.1117G>A	p.Gly373Arg	HOM	Missense	NA	0.00002392	0.00	D	D	D	25.6

1) Reference transcript of *TTC12* is NM_017868.4 and NP_060338.3.

2) Hom: homozygous.

3) PloyPhen-2: D, damaging; P, possible damaging. MutationTaster: D, disease causing; N, polymorphism. PROVEAN: D, deleterious; N, neutral. CADD (GRCh37-v1.4) score: amino acid substitution is predicted damaging if the score is > 10. NA: not available.

### Damaging impact of *TTC12* variants

The three detected *TTC12* variants were either at low allelic frequencies or undetectable in the public population databases and our fertile controls (Table 1). The two identified frameshift variants (c.1467_1467delG and c.1139_1139delA) were predicted to induce premature termination codons and truncated proteins. The residue Gly in the 373 locus is highly conserved among different species, and the missense variant [c.1117G>A (p.Gly373Arg)] was predicted to be deleterious using *in silico* bioinformatics software (Figures 1B, C; Table 1). Moreover, the substitution of neutral Gly with basic Arg at the 373 locus created a new hydrogen bonding connection between Arg373 and Leu363, which was postulated to destroy normal protein function (Figure 1D). To further evaluate the functional impact of the identified *TTC12* variants *in vitro*, we transfected wild-type and three mutant *TTC12* vectors into HEK293T cells. Compared with the wild-type, the two frameshift *TTC12* mutants [c.1467_1467delG (p.Asp490Thrfs*14) and c.1139_1139delA (p.His380Profs*4)] resulted in the formation of a truncated protein, which impaired protein integrity and normal function. All three variants, including the missense mutant [c.1117G>A (p.Gly373Arg], showed decreased expression levels of TTC12 relative to those in the wild-type control, implying protein instability (Figure 1E). Altogether, all the identified candidate variants were categorized as pathogenic according to the ACMG standards and guidelines (Richards et al., 2015) (c.1467_1467delG: PVS1_Strong + PS3 + PM2 + PM3_Supporting + PP4, c.1139_1139delA: PVS1_Strong + PS3 + PM2 + PM3_Supporting + PP4, and c.1117G>A: PS3 + PM2 + PM3_Supporting + PP3 + PP4). Therefore, we speculated that this impaired function, reduced expression, and increased instability of TTC12 caused by the identified homozygous *TTC12* variants might be responsible for infertility in our subjects.

### Clinical phenotype of patients with the *TTC12* variants

**FIGURE 2:**
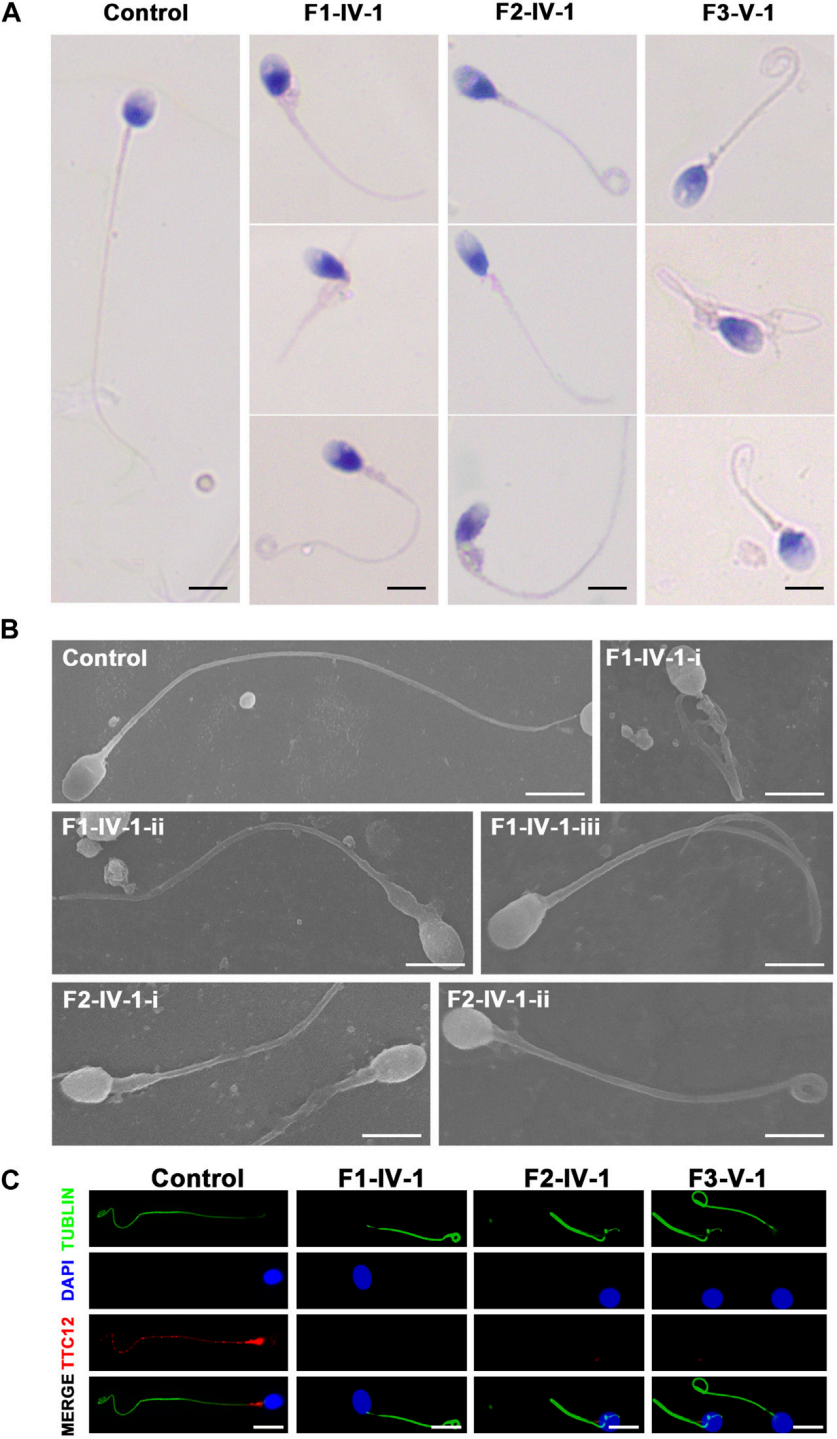
Spermatozoa morphology analysis of men with *TTC12* variants. **(A,B)** H&E and SEM examination results showed that men with homozygous *TTC12* variants displayed multiple MS and flagellar abnormalities in spermatozoa. MS, mitochondrial sheath. Scale bars: 5 μm. **(C)** TTC12 staining is expressed in the whole length of sperm flagella and was strongly concentrated in the mid-piece in control but almost absent in three affected individuals. Scale bars: 5 μm.

Semen parameters examined based on the WHO’s guidelines showed that sperm concentrations were normal in men with *TTC12* variants. However, the sperm was immotile in two probands (F1-IV-1 and F2-IV-1), and the sperm motility rate in the remaining individual (F3-V-1) was down to 1.36% (Table 2). Spermatozoa morphology was evaluated using H&E and SEM. Compared to the long and thread-like flagella observed in the control sperm, a remarkably increased number of spermatozoa in our probands presented multiple flagellar malformations, including absent, short, coiled, angulation, and irregular tails (Figures 2A, B; Table 2). Angulation and coiled flagella were the most frequently observed defects in the spermatozoa of the three patients (Figures 2A, B; Table 2).

**TABLE 2:** Spermatozoa examination of men with homozygous *TTC12* mutants.

Human subject	F1-T00580-IV-1	F2-T00715-IV-1	F3-T00725-V-1	Reference value^a^
Semen parameter				
Volume (mL)	4.4	5.0	2.1	>1.5
Sperm concentration (10^6^/mL)	27.97	114.51	23.15	>15.0
Motility (%)	0	0	1.36	>40.0
Progressive motility (%)	0	0	0	>32.0
Sperm flagellar morphology^b^				
Absent flagella (%)	10.5	5.7	8.3	<5.0
Short flagella (%)	10.6	7.9	4.3	<1.0
Coiled flagella (%)	30.3	25.4	40.4	<17.0
Angulation (%)	25.0	24.6	17.0	<13.0
Irregular caliber (%)	8.7	2.6	10.9	<2.0
Sperm mid-piece morphology				
Normal mid-piece (%)	39.9	39.7	45.2	>88.6
Abnormal mid-piece (%)	60.1	60.3	54.8	<11.4

^a^ Lower and upper reference limits according to the World Health Organization standards and the distribution ranges of morphologically abnormal spermatozoa observed in fertile individuals.

^b^ At least 200 spermatozoa were observed for morphological analysis.

The three subjects with *TTC12* variants also had a mild PCD-related nasosinusitis phenotype, which is a respiratory cilia impairment. However, they declined to undergo further examination of the concentrations of nasal NO, an abnormal ciliary beat frequency, and/or the presence of ciliary ultrastructural defects.

The damaging impact of the three novel *TTC12* variants was evaluated by using immunofluorescence staining with the TTC12 antibody. Observation showed TTC12 was present throughout the control flagella and was strongly concentrated in the mid-piece, whereas it was absent or almost undetectable in the three mutant carriers (Figure 2C). The above findings indicated that TTC12 deficiency causes asthenoteratozoospermia by causing flagellar abnormalities.

### *TTC12* defects induced axoneme ODA and IDA loss and MS malformations

**FIGURE 3:**
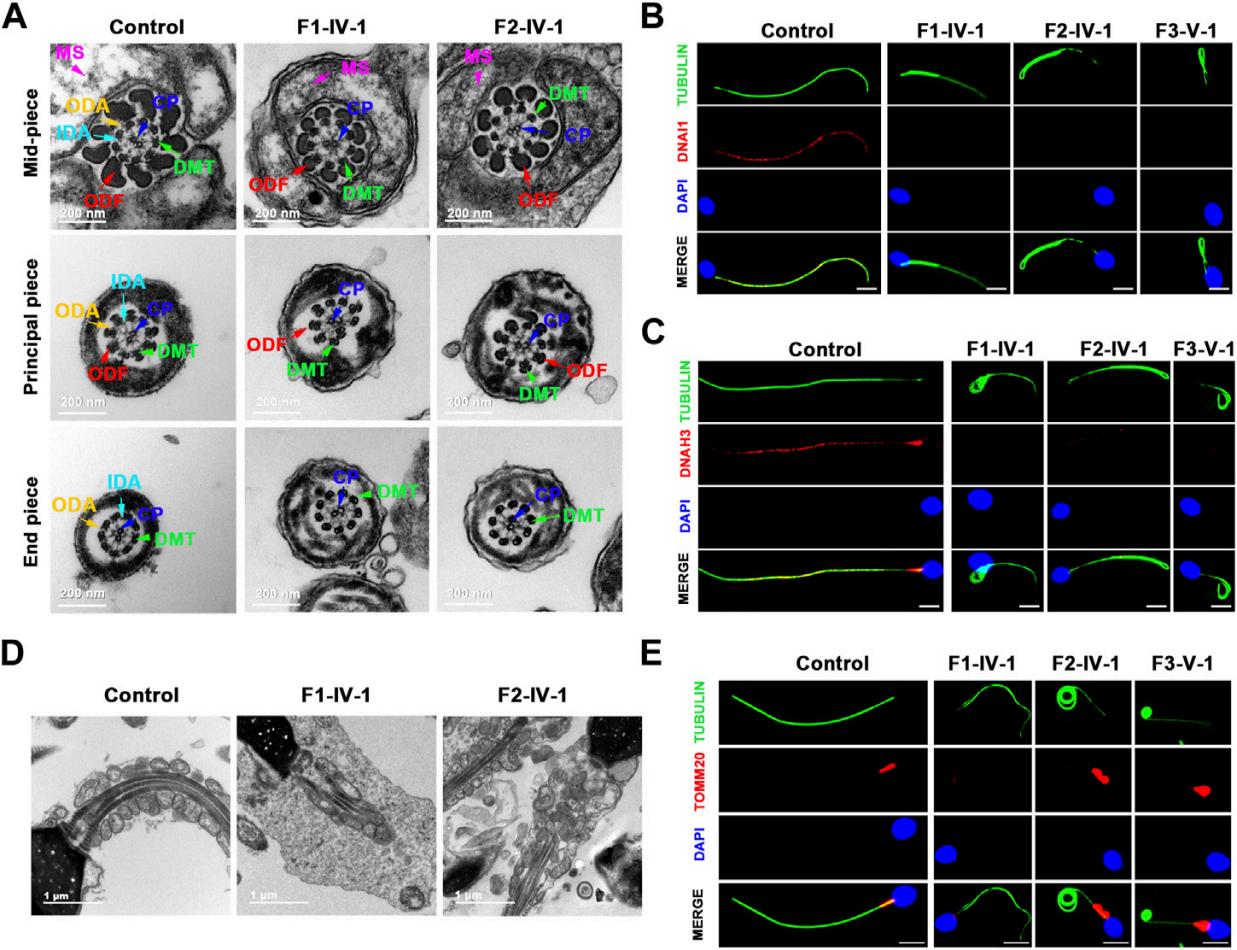
TTC12 defect associated with the absence of dynein arm complexes and mitochondrial sheath malformations. **(A)** Cross-sections of spermatozoa revealed the absence of dynein arm complexes in men with *TTC12* variants. ODA, outer dynein arms; IDA, inner dynein arms; DMT, doublets of microtubules; CP, central pair of microtubules; MS, helical mitochondrial sheath; and ODF, outer dense fibers. Scale bars: 200 nm. **(B,C)** Immunofluorescence staining of DNAI1 [red in **(B)**] and DNAH3 [red in **(C)**] showed that both were almost absent in sperm obtained from men carrying homozygous *TTC12* variants. Anti-α-tubulin (green) marked the sperm flagella. The nuclei of spermatozoa were DAPI-labeled (blue). Scale bars: 5 μm. **(D)** Longitudinal sections of sperm flagella showed infertile men with TTC12 defects, who had very thin and reduced number of mitochondria and absent annulus. Superfluous and/or disorganized mitochondria and excess cytoplasmic residues were clumped around the MS. Scale bars: 1 μm. **(E)** TOMM20 (red) presented abnormal staining with missing, distorted, and extended sperm flagellar mid-piece in the probands. DAPI present the nuclei of sperm (blue). Anti-α-tubulin showed flagella (green). Scale bars: 5 μm.

We performed TEM to further explore the sperm flagellar axoneme ultrastructural defects of the *TTC12* variant-carrying subjects. Individuals with *TTC12* variants exhibited a complete absence of ODA and IDA (Figures 3A–C; Supplementary Figure S1). Moreover, TEM scanning of control spermatozoa showed mitochondria arranged in regular loops covering both the connecting piece and mid-piece of the sperm flagellum, whereas the mid-piece of *TTC12*-mutated spermatozoa displayed very thin and reduced number of mitochondria and absent annulus. Moreover, superfluous and/or disorganized mitochondria and excess cytoplasmic residue were clumped around the MS in *TTC12* variant carriers (Figures 3D, E). Immunofluorescence assay was utilized to verify the axoneme ODA and IDA loss, and MS malformations were observed by TEM. The abundance and distribution of DNAI1 and DNAH17 (markers of ODA), DNAH3 and DNAH10 (markers of IDA), and TOMM20 (a marker of MS) were evaluated. The results showed that DNAI1 and DNAH3 were almost undetectable in the spermatozoa flagella of individuals with *TTC12* variants, which is consistent with the discovery of axoneme ODA and IDA loss (Figures 3B, C; Supplementary Figure S1). Additionally, TOMM20 presented abnormal staining with missing, distorted, and extended sperm flagellar mid-piece in our probands (Figure 3E).

We also examined the abundance and distribution of SPAG6 (a marker of CP), GAS8 (a marker of N-DRC), and RSPH1 (a marker of radial spoke). SPAG6, GAS8, and RSPH1 were almost consistent with the observations in controls (Supplementary Figure S2), indicating that the central pairs, N-DRC, and radial spoke might not be directly affected by these *TTC12* variants, which was consistent with the results observed in TEM.

### ICSI treatment of *TTC12*-associated male infertility

ICSI treatment was applied to male infertility and asthenoteratozoospermia caused by *TTC12* variants. The results of ICSI therapy revealed that the formation rates of two-cell embryos and eight-cell embryos were 88.9% and 77.8%, 87.5% and 25%, and 92.3% and 23.1% in families 1, 2, and 3, respectively (Table 3). Two out of three couples received embryo-transfer therapy and achieved good clinical outcomes (Table 3), suggesting that male infertility resulting from *TTC12* variants can be overcome by ICSI.

**TABLE 3:** Clinical results of ICSI therapy.

Subject	F1-IV-1	F2-IV-1	F3-V-1
Male age (years)	33	31	27
Female age (years)	28	29	27
Number of ICSI cycles	1	1	1
Number of oocytes injected	9	8	13
Number (and rate) of fertilized oocytes	9 (100%)	8 (100%)	13 (100%)
Number (and rate) of cleavage embryos	8 (88.9%)	7 (87.5%)	12 (92.3%)
Number (and rate) of eight cells	7 (77.8%)	2 (25.0%)	3 (23.1%)
Number of transfer cycles	1	1	NA
Number of embryos transferred per cycle	2	2	NA
Clinical pregnancy rates	100%	100%	NA
Delivered babies	1	2	NA

NA, not available.

## Discussion

The present study showed that biallelic loss-of-function variants in *TTC12*, a gene involved in axonemal dynein complex assembly and spermatozoon flagellar assembly, are associated with the asthenoteratozoospermia phenotype, characterized by multiple abnormalities of flagella, absence of dynein arm complexes, and MS malformations.

*TTC12* is located at 11q23.2 and encodes an evolutionarily conserved protein of the *TTC12* family consisting of 705 amino acids. A previous study showed that *TTC12* plays a cytoplasmic role in dynein arm assembly and/or transport, and defects in *TTC12* cause PCD (Thomas et al., 2020). In this study, we identified novel homozygous variants of *TTC12*, including two frameshifts and one missense variant, in three unrelated asthenoteratozoospermia-affected men. These three variants exhibited low allelic frequencies or were undetectable in the general public population. We found that the variants led to impaired stability of TTC12 in HEK293T cells as well as in patients’ spermatozoa. The previous study included two unrelated infertile male adults from Turkey and Europe with *TTC12* mutations. However, solid TTC12 defects that cause asthenoteratozoospermia and male infertility in the Chinese population have not been reported. Thus, our genetic and experimental study convincingly identified loss-of-function variants of *TTC12* in asthenoteratozoospermia-affected individuals in a Chinese infertile cohort, which confirmed the contribution of *TTC12* to male infertility.

Our study showed that spermatozoa from *TTC12*-variant carriers exhibited impaired flagellar motility and deficiency of ODA and IDA, consistent with the previous observations in two unrelated infertile male adults with homozygous variants in *TTC12*. However, sperm morphology examination revealed additional multiple morphological abnormalities of the sperm flagella (MMAF) feature. We speculated that the phenotypic heterogeneity and severity of asthenoteratozoospermia in cases with *TTC12* mutants may be associated with different variant-type loci and ethnic origins.

Notably, severe MS malformations in sperm flagella were observed in the *TTC12* variant subjects, including very thin and reduced number of mitochondria and absent annulus in the mid-piece. Moreover, superfluous and/or disorganized mitochondria and excess cytoplasmic residue were clumped around the MS indicating that insufficient energy generation due to MS disorganization might play a vital role in sperm motility reduction in these patients. The MS is a unique double-helical structure located in the mid-piece, where the mitochondria are tightly arranged around the axoneme. Mitochondria are involved in providing energy for flagellar motility, and morphological malformation and dysfunction of the MS might impair sperm motility, which consequently induces asthenoteratozoospermia and diminishes fertility. Previous studies have shown that defects in several genes, such as *Gykl1* and *Gk2*, cause MS abnormalities and asthenoteratozoospermia in mice (Chen et al., 2017). In addition, human asthenoteratozoospermia induced by variants of *DNHD1* (Tan et al., 2022), *CFAP58* (He et al., 2020), and *CFAP65* (Wang et al., 2019) was associated with MS defects. We noticed that these MS malformation-related protein functions varied significantly, suggesting that MS malformations might be a common feature of asthenoteratozoospermia. However, MS defects have not been fully described in most MMAF-affected cases, and the underlying molecular mechanisms remain to be elucidated. Therefore, further studies, such as *in vitro* and *in vivo* functional experiments using *TTC12*-knockout/knockin mouse models, should be conducted to shed light on the molecular mechanisms underlying spermatozoon flagellar abnormalities.

As expected, further examination of the three asthenoteratozoospermia-affected individuals with *TTC12* mutants revealed a mild PCD-related nasosinusitis phenotype, a respiratory cilia impairment that was usually ignored when these individuals requested infertility-related counseling. Our results confirmed that *TTC12* variants are related to PCD, suggesting that patients with asthenoteratozoospermia should pay attention to cilia-related phenotypes when they undergo infertility treatment.

Clinically, ICSI is a common strategy for conception in men with asthenoteratozoospermia-induced infertility. However, in previous studies, clinical outcomes have been varied greatly with different pathogenic genetic etiologies. For example, individuals with asthenoteratozoospermia caused by the *DNHD1* (Tan et al., 2022), *CFAP58* (He et al., 2020), and *CFAP47* (Liu et al., 2021) variants exhibited good clinical outcomes, whereas men with *CEP135* (Sha et al., 2017) and *CEP128* (Zhang et al., 2022) defects were unable to conceive. We noticed that men harboring *TTC29* variants achieved satisfactory ICSI outcomes, implying that homozygous *TTC12-*affected asthenoteratozoospermia can be overcome by ICSI. Subsequent investigations showed that two out of three female partners of the *TTC12* variant subjects successfully conceived, suggesting that ICSI therapy can be recommended for cases of *TTC12*-associated asthenoteratozoospermia. In particular, none of the female partners carried a pathogenic variation of the *TTC12* gene, indicating that their offspring are unlikely to be affected with PCD and asthenoteratozoospermia. Our findings provide informative insight for clinical genetic and reproductive counseling for asthenoteratozoospermia-affected individuals.

In conclusion, we found three infertile men carrying homozygous *TTC12* variants in a cohort of 314 asthenoteratozoospermia-affected Chinese cases. This report showed convincing *TTC12* pathogenic variants that cause asthenoteratozoospermia by causing dynein arm complex defects and mitochondrial sheath malformations in the flagellum, and TTC12 deficiency-related infertility can be overcome using ICSI technology.

## Data Availability

The original contributions presented in the study are included in the article/Supplementary Material, further inquiries can be directed to the corresponding authors.
