# Supplementary material for: Novel homozygous variants in TTC12 cause male infertility with asthenoteratozoospermia owing to dynein arm complex and mitochondrial sheath defects in flagella
Source: Front Cell Dev Biol. 2023 Jun 1;11:1184331. doi: 10.3389/fcell.2023.1184331 (PMC10267457; doi:10.3389/fcell.2023.1184331)
Supplement: Supplementary file 1 [file DataSheet1.DOCX]

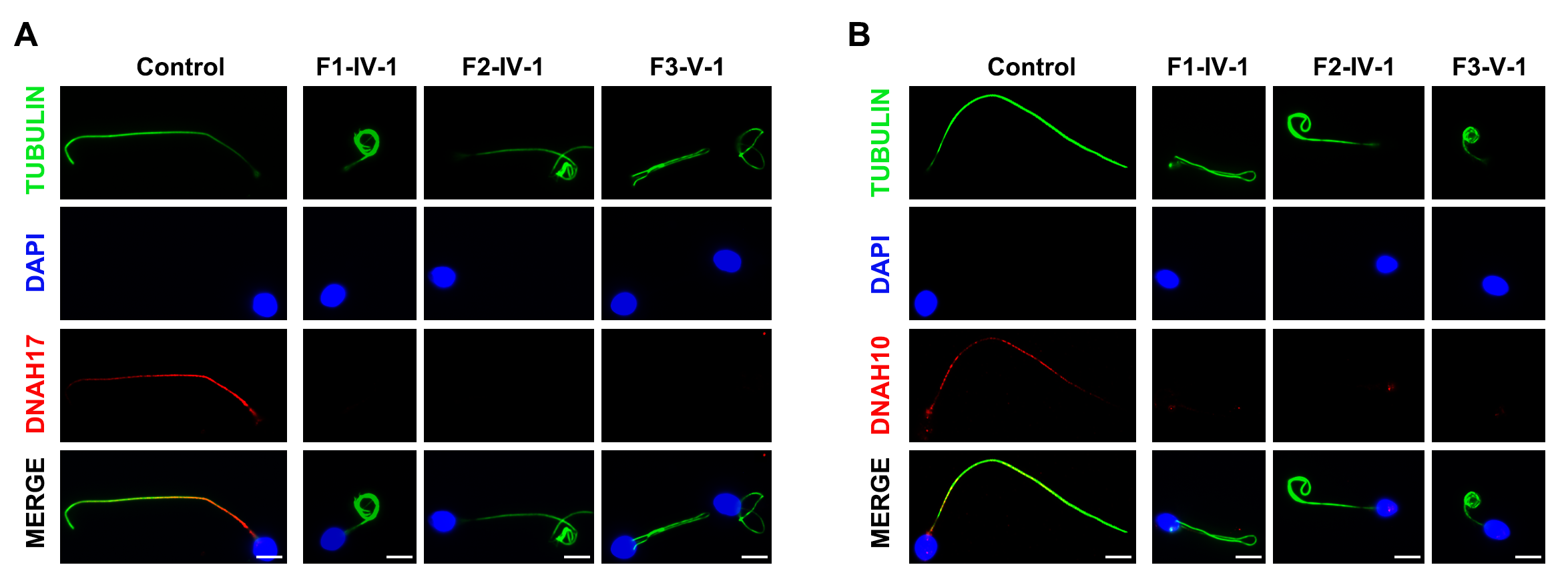


**Figure S1. Immunofluorescence staining of** **DNAH17 and DNAH10 in spermatozoa from control and men with *TTC12* variants.**

(A-B) DNAH17 and DNAH10 immunofluorescence assays of human sperm. DNAH17 (red in A), and DNAH10 (red in B) showed normally location along the sperm flagella of control, while both were almost absent in sperm obtained from men carrying homozygous *TTC12* variants. Anti-α-tubulin (green) marked the sperm flagella. The nuclei of spermatozoa were DAPI-labeled (blue). Scale bars: 5 μm.


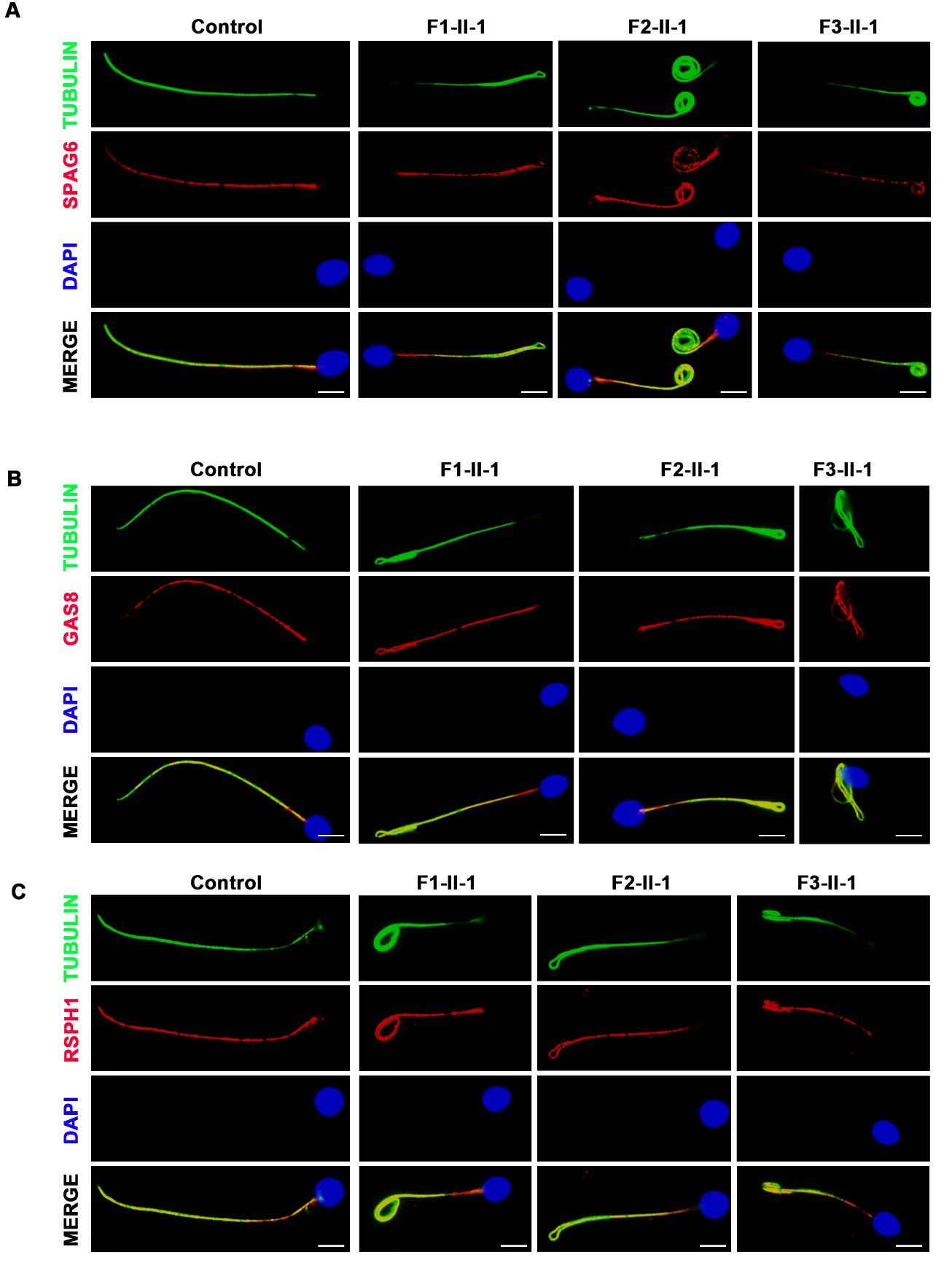


**Figure S2. Immunofluorescence staining of** **SPAG6, GAS8, and RSPH1.**

(A-C) SPAG6, GAS8, and RSPH1 immunofluorescence assays of human sperm. SPAG6 (red in A), GAS8 (red in B), and RSPH1 (red in C) showed normally location along the sperm flagella of control and men with homozygous TTC12 variants, suggesting CP, N-DRC and radial spoke were not directly affected. Anti-α-tubulin (green) and DAPI (blue) represented the sperm flagella and the nuclei of spermatozoa, respectively.

| **Table S1. Primers used for amplification and verification of *TTC12* variants** | | | |
| --- | --- | --- | --- |
| **Gene^1^** | **Exon** | **Primer sequence (5′-3′)** | **Tm^2^(°C)** |
| TTC12-13F | 13 | GACCCAGCAGTCTGGTTCT | 58 |
| TTC12-13R |  | GATAGGAGGGGAGACAGGCAT |  |
| TTC12-17F | 17 | GACAGTCCTTCCAGCCAACC | 58 |
| TTC12-17R |  | CATGCCCTCTTCTACCTTCG |  |

1 F, Forward primer; R, Reverse primer.

2 Tm, The annealing temperature.

| **Table S2. Antibodies used for immunofluorescence assay** | | | |
| --- | --- | --- | --- |
| **Name** | **Company** | **Catalog No.** | **Dilution** |
| rabbit polyclonal anti-TTC12 | Sigma | HPA038543 | 1:100 |
| rabbit polyclonal anti-TOMM20 | Proteintech | APR10507G | 1:600 |
| rabbit polyclonal anti-DNAI1 | Bioworld | BS90420 | 1:100 |
| rabbit polyclonal anti-DNAH10 | Bioss | bs-11022R | 1:100 |
| rabbit polyclonal anti-DNAH17 | Proteintech | 24488-1-AP | 1:100 |
| rabbit polyclonal anti-DNAH3 | Shanghai Youke Biotechnology | Co.Ltd | 1:100 |
| rabbit polyclonal anti-SPAG6 | Sigma | HPA038440 | 1:200 |
| rabbit polyclonal anti-GAS8 | Sigma | HPA041311 | 1:100 |
| rabbit polyclonal anti-RSPH1 | Sigma | HPA017382 | 1:100 |
| monoclonal mouse anti-α-tubulin | Sigma | T5168 | 1:1000 |
| Alexa Fluor 488 anti-mouse IgG | Invitrogen | A21121 | 1:1,000 |
| Alexa Fluor 555 anti-rabbit IgG | Invitrogen | A32732 | 1:1,000 |
